# A Unique Case of Vascular Variations in the Upper Limbs: A Brachioradial Artery and Bilateral Persistent Median Arteries With Incomplete Superficial Palmar Arches

**DOI:** 10.7759/cureus.70634

**Published:** 2024-10-01

**Authors:** Carl W Lee, Jillian K Vlasak, Stuart G Atwood, Yun Tan, Daniel T Daly

**Affiliations:** 1 Department of Surgery, Center for Anatomical Science and Education, Saint Louis University School of Medicine, St. Louis, USA; 2 Department of Occupational Health and Occupational Therapy, Saint Louis University Doisy College of Health Sciences, St. Louis, USA

**Keywords:** anatomy & physiology, cadaver case report, incomplete palmar arch, persistent median artery, vascular variation

## Abstract

Numerous vascular variations were observed in the upper limbs of a 109-year-old female donor to the Gift Body Program of Saint Louis University School of Medicine. Variations in the right upper limb included the presence of a brachioradial artery (BRA), persistent median artery (PMA), and an ulnar-dominant incomplete type B superficial palmar arch (SPA). In the left upper limb, the brachial artery bifurcated normally into the ulnar artery (UA) and radial artery (RA). However, a more developed palmar type of PMA was observed, replacing much of the palmar circulation typically supplied by the superficial palmar branch of the RA, which existed only as a small branch anastomosing with the PMA to form a rare arcus medianoradialis-type SPA. Both PMAs arose from their respective UAs distal to the origin of the common interosseous arteries. The left PMA pierced the median nerve (MN) as it descended the forearm. The extent of these variations together presents a unique case of vascularity in the upper limbs. Knowledge of these variations is pertinent to MN compression pathologies as well as procedures involving the upper limb.

## Introduction

The brachial artery begins at the lower border of the teres major muscle as the direct continuation of the axillary artery. Three branches important to distal circulation typically originate from the brachial artery: the deep brachial artery, and both the superior and inferior ulnar collateral arteries. These three branches serve as the vascular supply of the brachium and elbow region, including bone and soft joint tissue. Typically, the brachial artery passes through the cubital fossa before bifurcating into the radial artery (RA) and the ulnar artery (UA). The RA continues distally and laterally after giving off the radial recurrent artery while the UA continues deep and medial, giving off the posterior and anterior ulnar recurrent arteries and the common interosseous artery. The common interosseous artery bifurcates into the anterior interosseous artery and the posterior interosseous artery, which then gives off the interosseous recurrent artery. The anterior and posterior interosseous arteries serve their respective compartments of the antebrachium. The anterior/posterior ulnar recurrent, radial recurrent, and interosseous recurrent arteries serve as the antebrachial components of collateral elbow joint circulation, with the interosseous recurrent supplying the elbow joint itself.

Early fetal development of the arterial patterns in the upper limb is dominated by the presence of the axial artery, which serves as the primary blood supply to the limb bud as it develops. During the first trimester of gestation, the median artery develops from the axial system, and along with the anterior interosseous artery, is the major blood supply to the hand. The median artery usually undergoes apoptosis and regresses during the eighth week of intrauterine development as the UA and RA arise to take over palmar circulation, thus it is often considered a transitory embryonic structure [1].

In some individuals, the median artery persists into adulthood as a main vascular supply to the median nerve (MN), adjacent structures, or the hand. This remaining embryological vessel, referred to as the persistent median artery (PMA), can be categorized as one of two types: palmar or antebrachial. The morphology of the antebrachial type does not cross the wrist and is far more delicate than the larger caliber palmar type, which traverses the carpal tunnel and contributes to the arterial supply of the hand [1,2]. The most common origin of the palmar type is seen originating at the caudal angle of the UA and common interosseous artery, while the more common antebrachial type typically arises directly from the anterior interosseous artery [3].

One meta-analysis that investigated the reported prevalence of the PMA in cadaveric, surgical, and medical imaging studies found the antebrachial type to be more common (34%) than the palmar type (8.6%) [2]. Due to the frequent presence of the antebrachial type of PMA, some authors consider it a “normal” feature rather than a variation in adults [4]. Twenty-two studies among adults and infants reported a pooled bilaterality prevalence rate of 20.4% for the palmar type of PMA with no significant difference observed between sexes [2]. However, age had a considerable effect on the prevalence of the palmar PMA; 18.6% of infants presented with a palmar PMA, while only 7.5% of adults presented [2]. The higher prevalence of the palmar PMA in infants is explained by the likelihood of continued postnatal development and regression of this artery at a later stage of life [1]. In the hand, the superficial palmar arch (SPA) is typically found deep to the palmar aponeurosis and superficial to the tendons of the flexor digitorum superficialis [5]. Normally, the primary contribution of the SPA is the superficial UA, which anastomoses with the superficial palmar branch of the RA to form an arching arterial structure [5]. The SPA typically gives off three common palmar digital arteries, which bifurcate into proper palmar digital arteries, each supplying half of two adjacent digits [5]. Additionally, a singular palmar digital branch from the SPA arises to supply the medial side of the fifth digit [5]. The SPA is regularly observed as complete and less frequently as incomplete. A complete arch, formed by both ulnar and radial contributions, was reported in 58%-80% of sampled cadaveric donors [6,7]. An incomplete arch of the SPA describes cases in which the ulnar and radial contributions to palmar circulation do not form an anastomotic connection [8].

## Case presentation

**Figure 1:**
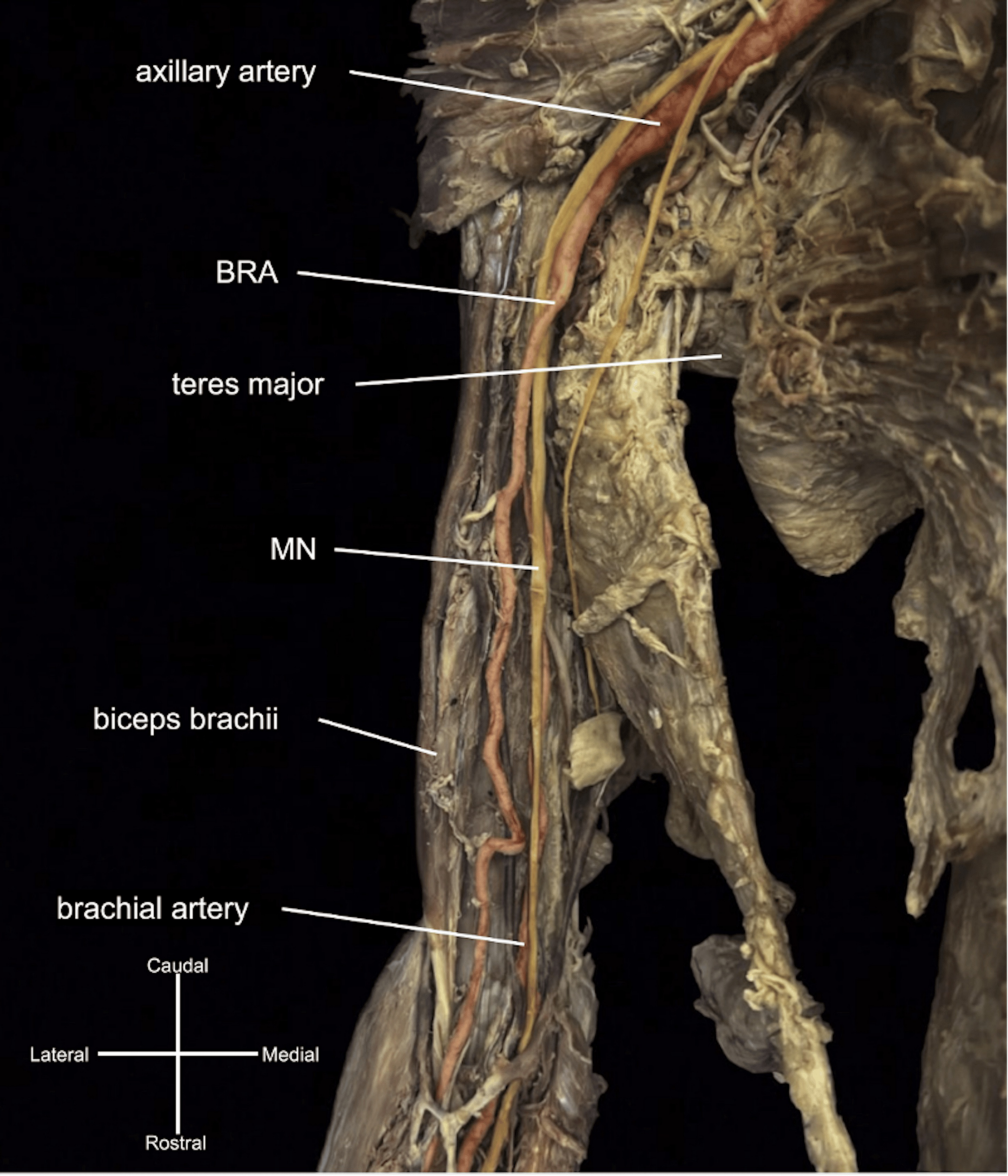
Atypical brachioradial branch of the right arm. A brachioradial artery (BRA) originated near the transition between axillary and brachial artery muscle. The BRA crossed over the median nerve (MN), continued distally and laterally, superficial to the biceps brachii muscle near the cubital fossa. The brachial artery traveled deep to the biceps brachii and the MN in the anterior compartment of the arm.

**Figure 2:**
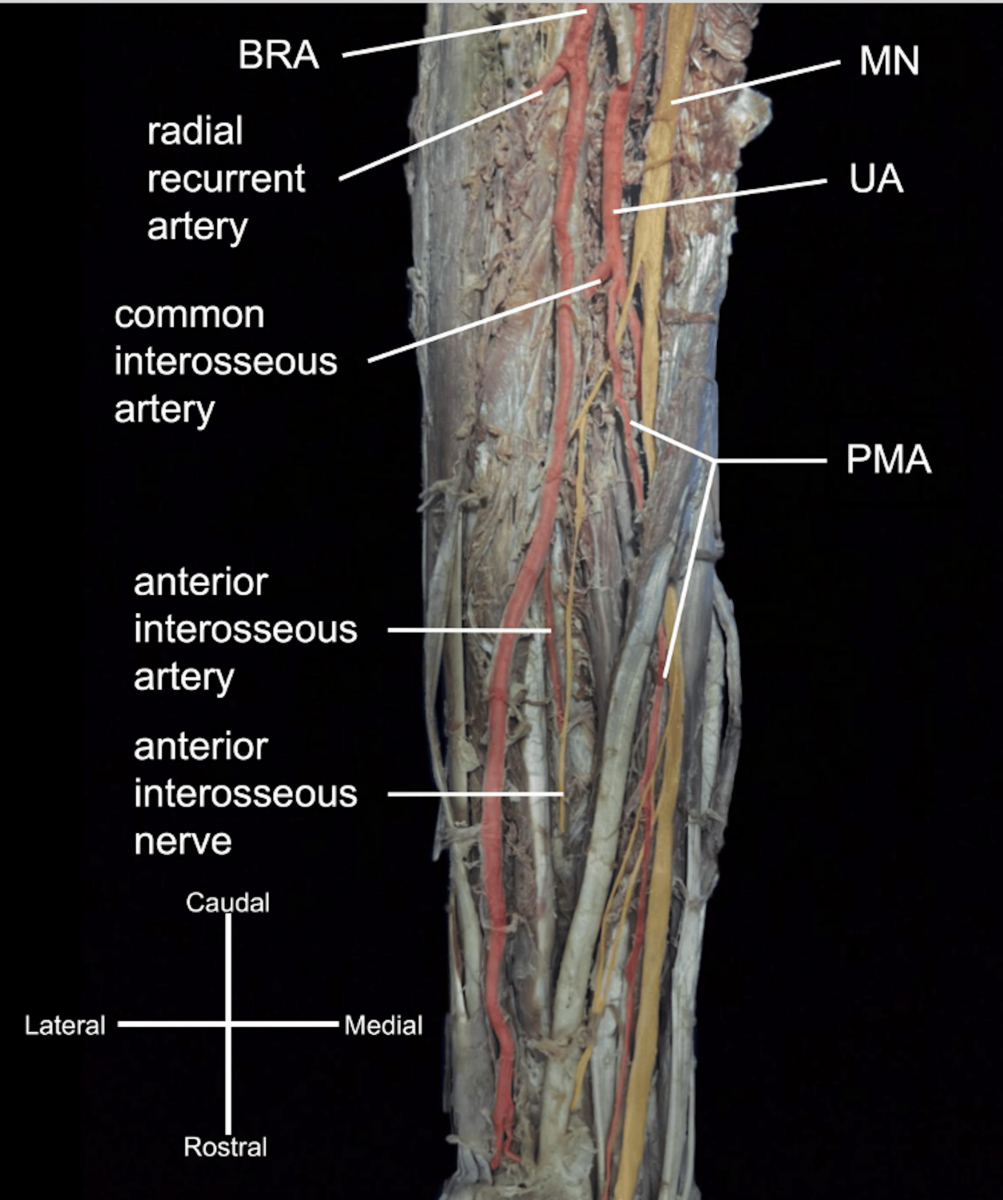
Arterial branching in the right antebrachium. After passing through the cubital fossa, the brachioradial artery (BRA) gave off the radial recurrent artery. The persistent median artery (PMA) branched from the ulnar artery (UA) slightly distal to the origin of the common interosseous artery and crossed superficial to the anterior interosseous nerve. The PMA supplied the median nerve (MN) as it continued past the wrist deep to the flexor retinaculum.

Complex bilateral variations of the arterial branching patterns in the upper extremities of a 109-year-old female cadaver were observed during routine dissection. The individual’s provided medical history did not offer any pertinent details regarding these observed variations. On the right, an atypical brachioradial artery (BRA) originated near the transition between the axillary artery and the brachial artery (Figure 1). The BRA crossed over the MN in the axilla near the level of the teres major, continued laterally, crossed superficial to the biceps brachii tendon near the cubital fossa, and then gave off its typical radial recurrent artery at the elbow joint. The brachial artery continued medially, deep to the biceps brachii near the cubital fossa, continuing as the UA after passing the cubital fossa to give off typical branches of the UA before giving off the PMA (Figure 2).

**Figure 3:**
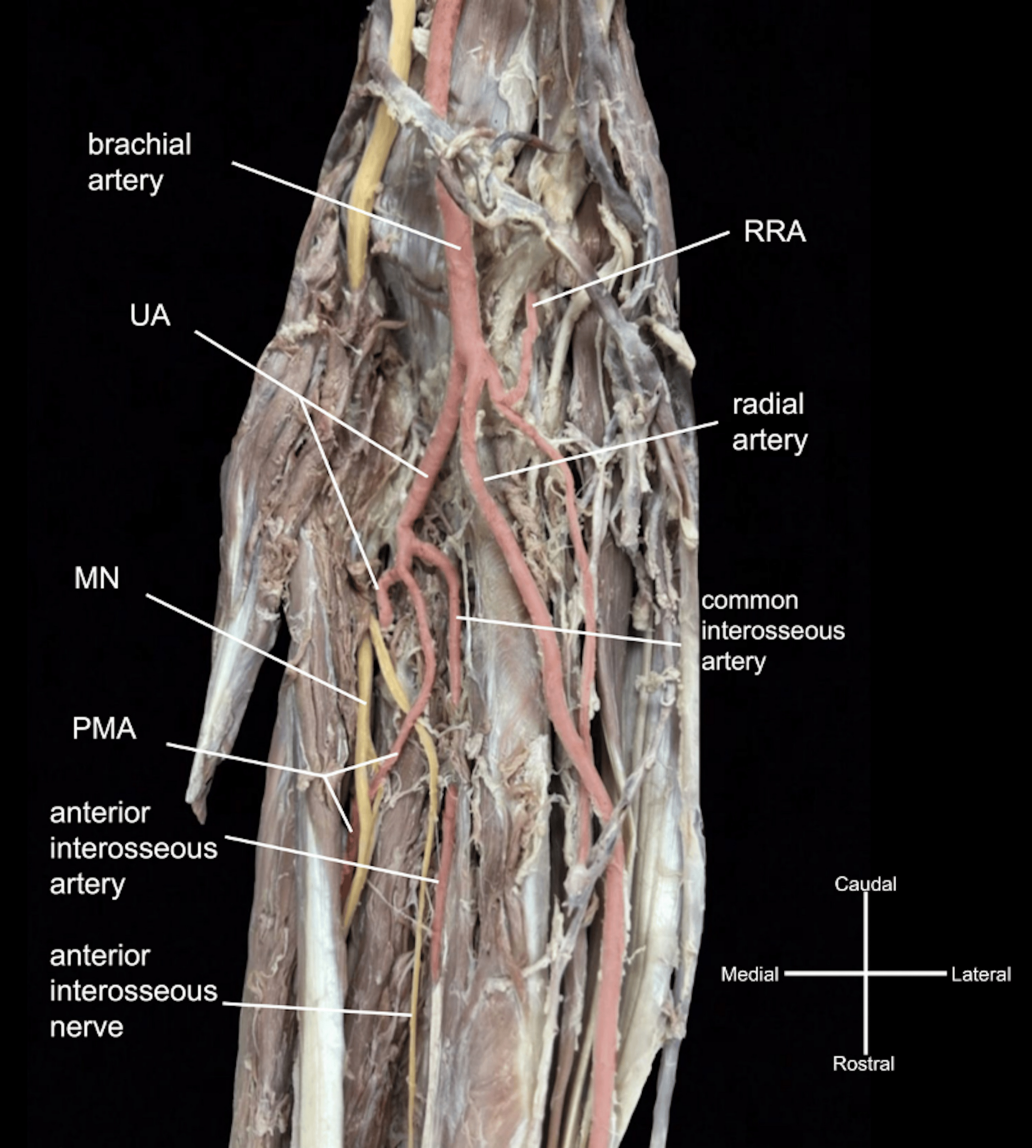
Arterial branching in the proximal left antebrachium. After passing through the cubital fossa, the brachial artery branched into the ulnar artery (UA), radial artery (RA), and radial recurrent artery (RRA), which gave off a significant muscular branch. Before continuing deep and medially, the UA gave off the common interosseous artery and an atypical persistent median artery (PMA), which crossed superficially to the anterior interosseous nerve before piercing the median nerve (MN).

**Figure 4:**
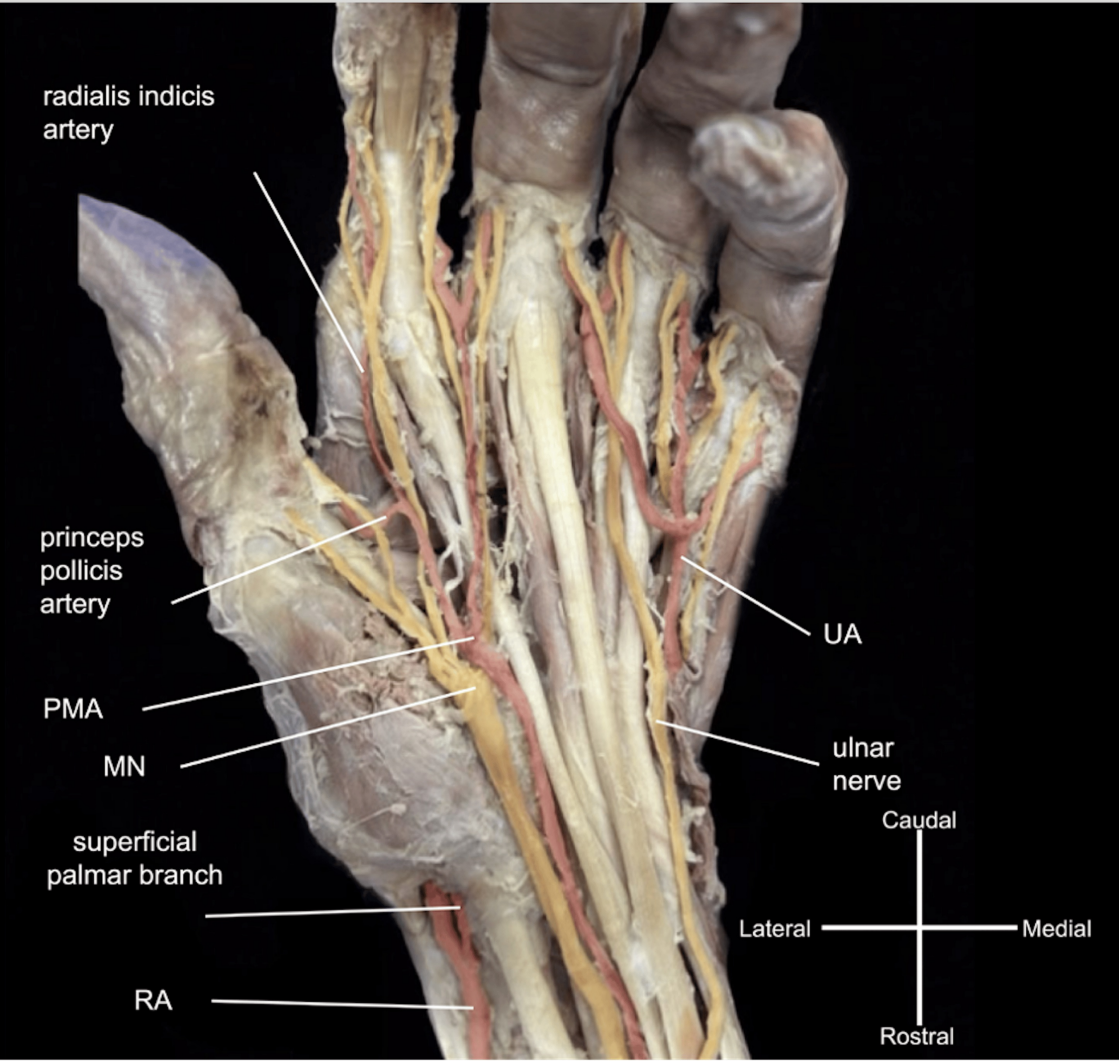
Left superficial palmar arch its branches. Similar to incomplete arch type C: The persistent median artery (PMA) formed a major contribution to the incomplete superficial palmar arch (SPA) with the superficial palmar branch of the radial artery (RA). Similar to common palmar digital branching type 3 or 5: UA gives off two common palmar digital arteries that bifurcate to supply the medial 2½ digits. The PMA gave off one common palmar digital artery which bifurcated into two proper palmar digital arteries to supply the lateral side of the third digit and the medial side of the index finger. Additionally, the princeps pollicis and radialis indicis arteries branched from the PMA as well.

The typical course of the axillary artery and branching pattern of the brachial artery were observed in the left brachium. The left brachial artery bifurcated into the RA and UA in the antebrachium. The RA provided a typical branching pattern (Figure 3) and displayed a relatively small superficial palmar branch (Figure 4).

The UA branching was bilaterally uniform, giving off the expected recurrent branches and the common interosseous artery. PMAs on both sides were observed originating from the UA, slightly distal to the common interosseous artery origins (Figures 2, 3). The bilateral PMAs supplied their respective MNs, with the left PMA piercing the MN, as they accompanied them through the anterior antebrachial compartments, the carpal tunnel, and the left continuing into the hand (Figures 2-4).

**Figure 5:**
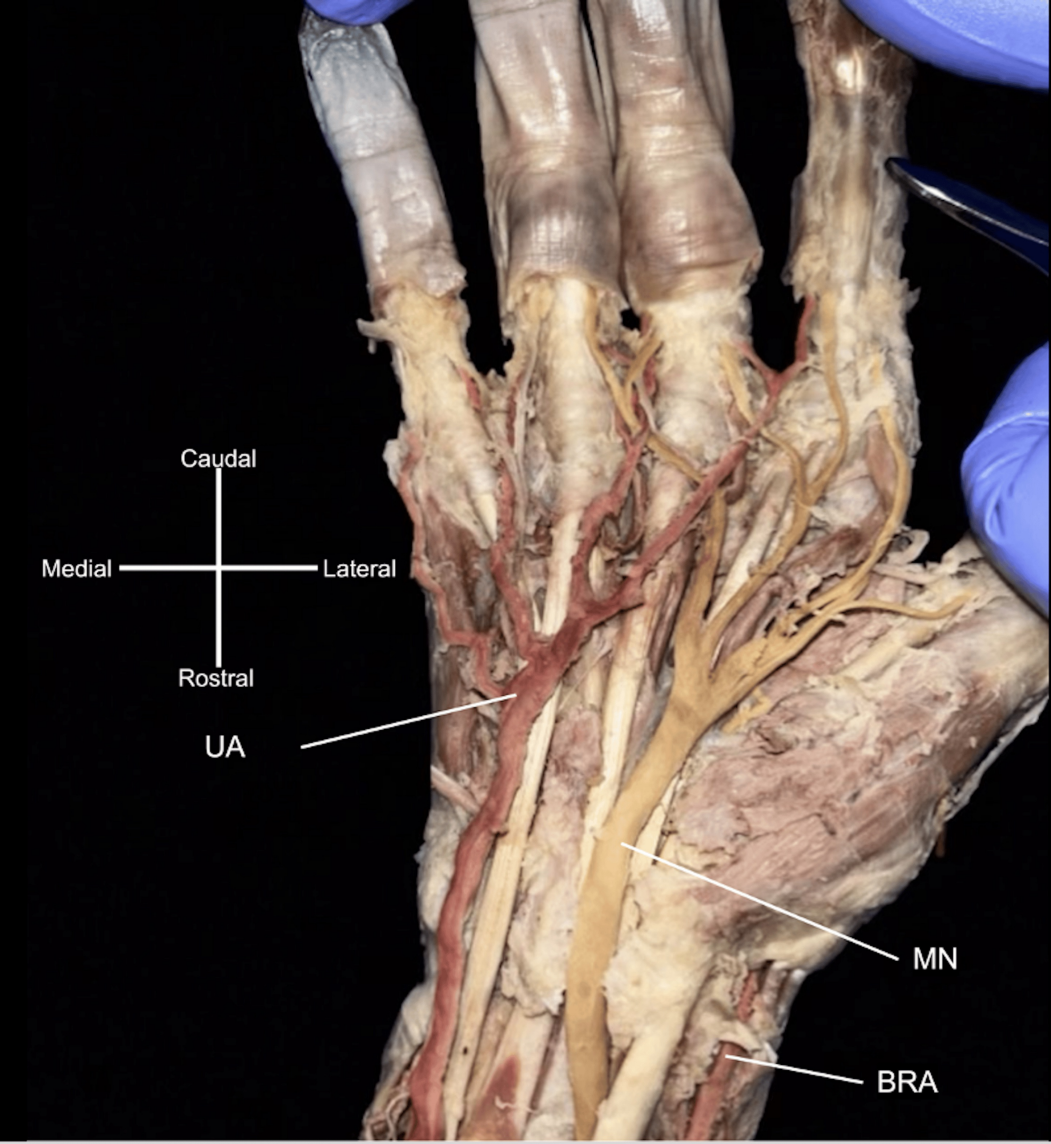
Right superficial palmar arch and its branches. Incomplete arch type B: The ulnar artery (UA) formed an incomplete superficial palmar arch (SPA) and the brachioradial artery (BRA) passed into the thenar compartment without giving off the superficial palmar branch. Common palmar digital branching type 2: The SPA gives off three common palmar digital arteries that bifurcate into proper palmar digital arteries to supply the medial 3½ digits. The BRA gives off radialis indicis and princeps pollicis arteries to supply the lateral side of the second digit and first digit, respectively.

In the palmar regions, incomplete SPAs were seen bilaterally. In the right palm, the UA component of the incomplete SPA supplied the medial side of the digit 2 and digits 3-5 completely, while the superficial palmar branch of BRA appeared to terminate in the thenar compartment (Figure 5). The right PMA terminated deep into the flexor retinaculum and thus did not contribute to the SPA. The BRA displayed typical branches of the RA while traversing the dorsum of the hand, providing vascularity to digit 1 and the lateral side of digit 2 through the princeps pollicis artery and the radialis indicis artery, respectively, before contributing to the deep palmar arch.

The left palm showed that the UA contribution of an incomplete SPA supplied the medial side of digit 3 and digits 4-5 completely (Figure 4), while the superficial palmar branch of the RA extended through the thenar compartment to anastomose with the PMA. The left PMA supplied the remaining digits and branched ventrally into radialis indicis artery (RIA) and princeps pollicis artery (PPA) (Figure 4). The RA coursed toward the dorsum of the hand to its typical continuation as the deep palmar arch.

## Discussion

The high origin of the RA observed in this case, known as the BRA, has been reported in 9.2-20.3% of upper limbs [4,9,10]. Within cadavers possessing a BRA, the most common site of this vessel’s origin was seen from the upper third of the BA (65.4%) and less commonly from the axillary artery (23%) [9]. The BRA typically runs superficial to the MN along the arm and in many cases gives off the radial recurrent artery in the forearm (46%). It should be noted that some cases indicate the presence of an anastomotic connection between the BRA and BA (26.4%) and thus can be further classified based on its position to the bicipital aponeurosis in the cubital fossa [9]. This “cubital crossover” as it is known, which is estimated to occur in 45.4% of BRAs [10,11], does not occur in the current case.

The high bifurcation of the brachial artery is a relatively common occurrence and has recently been shown to result in a high failure rate and decreased functional patency of an arteriovenous (AV) fistula [12]. Because high bifurcation could be present in a significant number of patients and impact the efficacy of AV access, surgeons should use vascular mapping to evaluate the presence of a BRA and use the appropriate surgical technique. The BRA is also used in vascular, plastic, and reconstructive surgery; its unusual origin can present risks for transradial access because of its routine use for catheterization [10,11]. Additionally, the RA is the second most used artery in coronary bypass grafting [13]. With these implications, there is a risk of perforation due to the variable origin of the radial recurrent artery, or an existing cubital crossover.

The antebrachial PMA is a known anatomical variant occurring in 34.1% of the adult population, while the palmar type is less common, with a prevalence of 7.5%. Only 17.4% of palmar PMAs present bilaterally, with a prevalence of 1.3% in the adult population [2]. This case describes the rare presence of bilateral PMAs (one palmar and one antebrachial) in conjunction with the unilateral presence of a BRA and two distinct SPA morphologies.

The bilateral PMAs observed in this case were observed as palmar type on the left and antebrachial type on the right, but both limbs presented with incomplete SPAs. Based on a study of 650 cadaveric specimens, Coleman and Anson described the SPA as either a complete arch type A-E or an incomplete arch type A-D (Table 1) in addition to categorizing the common palmar digital artery branching patterns from the SPA as types 1-7 (Table 2) [7]. This system of categorizing the SPA and its branching pattern has been oft-cited and utilized to classify morphologies of arterial organization in the palm that are inconsistent with the classical understanding [1,5-7,9,14-20]. An alternative categorization system for the SPA, proposed by Lippert and Pabst in 1985, describes four types of complete SPA and five types of incomplete SPA. Under this system, a complete arch is defined as an anastomosis between any two vessels forming the SPA [21]. Notably, Lippert and Pabst include a description of a complete SPA, the arcus medianoradialis, that is not described by the Coleman and Anson system. The arcus medianoradialis is seen when the superficial RA and PMA anastomose to supply the lateral side of the hand and the ulnar artery alone supplies the medial side of the hand [21].

**Table 1:** Description of the Coleman and Anson categorization of complete and incomplete superficial palmar arch indicating the arteries involved in each type as well as their incidence.

	Type	Involved arteries	Incidence
Complete arch	A	Radial and ulnar artery	34.5%
	B	Ulnar artery only; supplies thumb and index finger	37.0%
	C	Ulnar and median artery	3.8%
	D	Radial, median, and ulnar artery	1.2%
	E	Mainly ulnar artery; anastomosis with deep palmar arch	2.0%
Incomplete arch	A	Radial and ulnar artery	3.2%
	B	Ulnar artery only; does not contribute to thumb and index finger supply	13.4%
	C	Ulnar and median artery	3.8%
	D	Radial, median, and ulnar artery	1.1%

**Table 2:** Description of the Coleman and Anson categorization of branching patterns of common palmar digital arteries from the superficial palmar arch as well as the incidences.

Type	Description	Incidence
1	Four common palmar digital arteries; the first eventually bifurcating into the arteries supplying the ulnar side of the thumb and radial side of the index finger. Arteries 2-4 exhibit regular branching	77.3%
2	(Typical presentation) Three common palmar digital arteries traveling to interdigital spaces 2-4 then bifurcating to supply between the ulnar side of the index finger and the radial side of the fifth digit	8.8%
3	Three common palmar digital arteries that exhibit normal branching but with an additional branch from the SPA anastomosing with or replacing princeps pollicus artery without supplying the radial side of the index finger	6.4%
4	Three common palmar digital arteries; the first eventually bifurcating into the arteries supplying the ulnar side of the thumb and radial side of the index finger. The second and third arteries travel to the second and fourth interdigital space, respectively, where they bifurcate normally	1.9%
5	Three common palmar digital arteries with the addition of the radialis indicis originating from the SPA	3.4%
6	Two common palmar digital arteries travel to the second and third interdigital space. There is no supply to the fourth interdigital space from the SPA	1.5%
7	Two common palmar digital arteries travel to the third and fourth interdigital space. There is no supply to the second interdigital space from the SPA	0.7%

The SPA of the right palm is characteristic of a type B incomplete arch described by Coleman and Anson, which is the most commonly observed incomplete SPA variation at 13.4% [7]. Additionally, the branching pattern of the palmar digital arteries in the right hand is best described as type 2, which was only observed in 8.8% of the specimens, despite being the “textbook” configuration [7]. The vascular arrangement on the left SPA does not fit cleanly into the Coleman and Anson alphanumeric classification, but it most closely resembles the incomplete arch type C classification, which has a reported prevalence of 3.8% [7]. In our case, the left SPA is best described by the arcus medianoradialis classification from Lippert and Pabst [21]. Furthermore, the distal branching from the left SPA is most similar to a type 3 or 5 digital artery branching pattern (Table 2), but the superficial branch of RA does not give off a branch to the first digit as it should in a type 3. There is also a conspicuous absence of a branch from SPA supplying the radial side of the index finger, as would be expected for a type 5 classification.

The presence of a PMA has been associated with several important clinical implications often involving MN compression and related syndromes. One such pathology, anterior interosseous nerve syndrome, is caused by either compression of the MN before anterior interosseous nerve branching, or a PMA that is intimately related to the anterior interosseous nerve as it crosses the forearm [9]. Both PMAs in this case cross superficially to the anterior interosseous nerve, which could result in paralysis of the flexor pollicis longus, the pronator quadratus, and the lateral half of the flexor digitorum profundus muscle, producing significant functional deficits [1]. Similarly, pronator teres syndrome can be associated with the PMA perforating the proximal part of the MN in the upper third of the antebrachium, namely, the part that is deep to the humeral head of pronator teres [4,20]. The presence of the PMA also increases the risk for entrapment of the MN as it passes through the carpal tunnel, particularly if thrombosis, a bifid MN, or an aberrant lumbrical is present to increase pressure deep to the flexor retinaculum [22].

The PMA by itself is not a cause of carpal tunnel syndrome, rather, the potential for thrombosis, calcification, or enlarged diameter poses an increased risk [14,17,22,23]. The PMA is typically variable in size and was shown to range from 1.5 to 2.0 mm at the distal rim of the flexor retinaculum in a study including four cadaveric samples [20]. In rarer cases, the PMA has been as large as 3.0 mm [17]. In our case, the left PMA measured 2.1 mm at the flexor retinaculum, while the right PMA measured 1.4 mm at the flexor retinaculum. The presence of a PMA measuring greater than a diameter of 2.0 mm was found to be the only cause of carpal tunnel syndrome in nine patients from two clinics in a study by Barfred et al. [24]. In the left palm of this unique case study, the PMA terminates as the PPA, RIA, and third common digital artery. A variation of this type is extremely rare, with only one other report of similar findings by Bataineh and Moqattash [15]. If a thrombus forms in a PMA which contributes to the SPA and thus entrapping the MN, conservative treatment with anticoagulation, thrombolysis, or more invasive surgical removal must be performed for the management of carpal tunnel syndrome [22].

This example demonstrates the PMA as a predominant blood supply to the radial side of the right palm. Therefore, in cases of arterial grafting, outcomes of RA harvesting may be impacted due to the insufficient diameter of a potentially hypoperfused RA. The diameter of the RA is said to be diminished if the PMA is well-developed [20]. Preoperative evaluation, such as CT angiography, can confirm the appropriate diameter of the RA and analyze the risk for ischemia in the presence of palmar circulation abnormalities [18]. Additionally, the presence or absence of a PMA should be noted in cases of graft tendon harvesting, as the palmaris longus tendon is typically in close topographical proximity to the distal course of the PMA [2,12].

Precautions should be taken before surgical procedures to identify and locate an unusual vessel of the upper limb, such as a BRA or PMA. The presence of these vulnerable vessels and the absence of the anastomotic connections of their typical varieties places patients at risk of ischemia and procedural complications. Techniques such as ultrasonography, color Doppler ultrasound, and magnetic resonance imaging have been used to locate vascular anomalies, particularly the PMA, for a variety of pathological conditions [14,15,17].

## Conclusions

The novel combination of a unilateral BRA, bilateral palmar PMAs with variable contribution to SPAs, and unique common digital artery branching from the SPA present a unique combination of variations that, when taken as a whole, have not been reported in a single individual. Awareness of these variations, particularly this unique set of variations, has clinical implications in physical medicine, surgery, and radiology.
